# Supplementary material for: Generation of gravity waves from thermal tides in the Venus atmosphere
Source: Nat Commun. 2021 Jun 17;12:3682. doi: 10.1038/s41467-021-24002-1 (PMC8211692; doi:10.1038/s41467-021-24002-1)
Supplement: Supplementary file 1 — Supplementary Information [file 41467_2021_24002_MOESM1_ESM.pdf]

Supplementary information

Generation of gravity waves from thermal tides in the Venus atmosphere

Sugimoto *et al*.

Contents of this file

Supplementary Note 1 to 6

Supplementary Figures 1 to 6

Supplementary Note 1

This supplementary information provides zonal mean fields for nominal and Qz cases (Supplementary Note 2), individual distributions of eddy kinetic energy E_k and potential energy E_p for the nominal and Qz cases (Supplementary Note 3), static stability and Richardson number for the nominal case (Supplementary Note 4), the vertical momentum flux and its mean zonal acceleration for the Qz case (Supplementary Note 5), and the coefficient of Rayleigh friction used as the sponge layer (Supplementary Note 6).

Supplementary Note 2 Zonal mean field

Supplementary Figure 1 shows latitude–height distributions of zonal mean zonal flow

and temperature deviation from its horizontal average obtained for nominal (a) and Qz (b) cases. Since we conducted high-resolution runs by restarting with different resolutions for 5 Earth years, the fast super-rotation of ~100 m s^{-1} with weak mid-latitude jets at the cloud-top (~70 km) level reached a quasi-equilibrium state for the nominal case. For Qz, because equatorward momentum transport due to thermal tides disappeared, stronger mid-latitudes jets were formed in the higher latitudes near the cloud-top compared with the nominal case. This is consistent with previous Venus GCMs without the thermal tides[1,2].

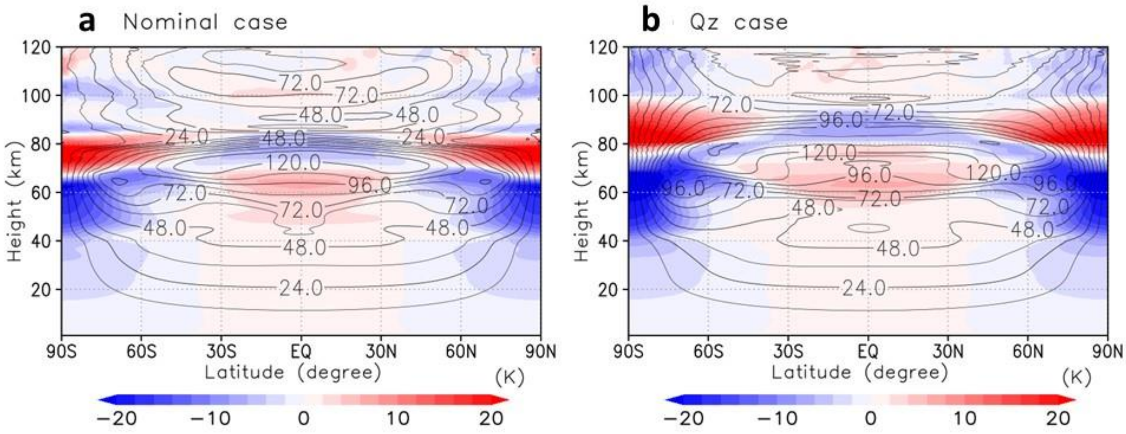

Supplementary Figure 1 Zonal mean fields. Panels **a**, **b** shows latitude–height cross-sections of zonal mean zonal flow (black contours, m s^{-1}) and temperature deviation from its horizontal average (colour shades, K) obtained for nominal (with thermal tides) and Qz (without thermal tides) cases, respectively. These distributions are averaged over 30 Earth days.

Supplementary Note 3 Eddy kinetic energy and potential energy

Supplementary Figure 2 shows eddy kinetic energy E_k and potential energy E_p in longitude–latitude cross-sections at the cloud-top level and longitude–height cross-sections at the equator for the nominal case. E_k was large especially in the mid-latitudes, which will be attributed partly to baroclinic waves. In addition, E_k and E_p had a wavenumber 2 component in the low-latitudes, which would be associated with thermal tides because this structure disappeared for Qz (Supplementary Fig. 3). Large amount of E_k and E_p were located at jet-exit regions formed by thermal tides as shown in the vertical section (Supplementary Figs. 2c and 2d).

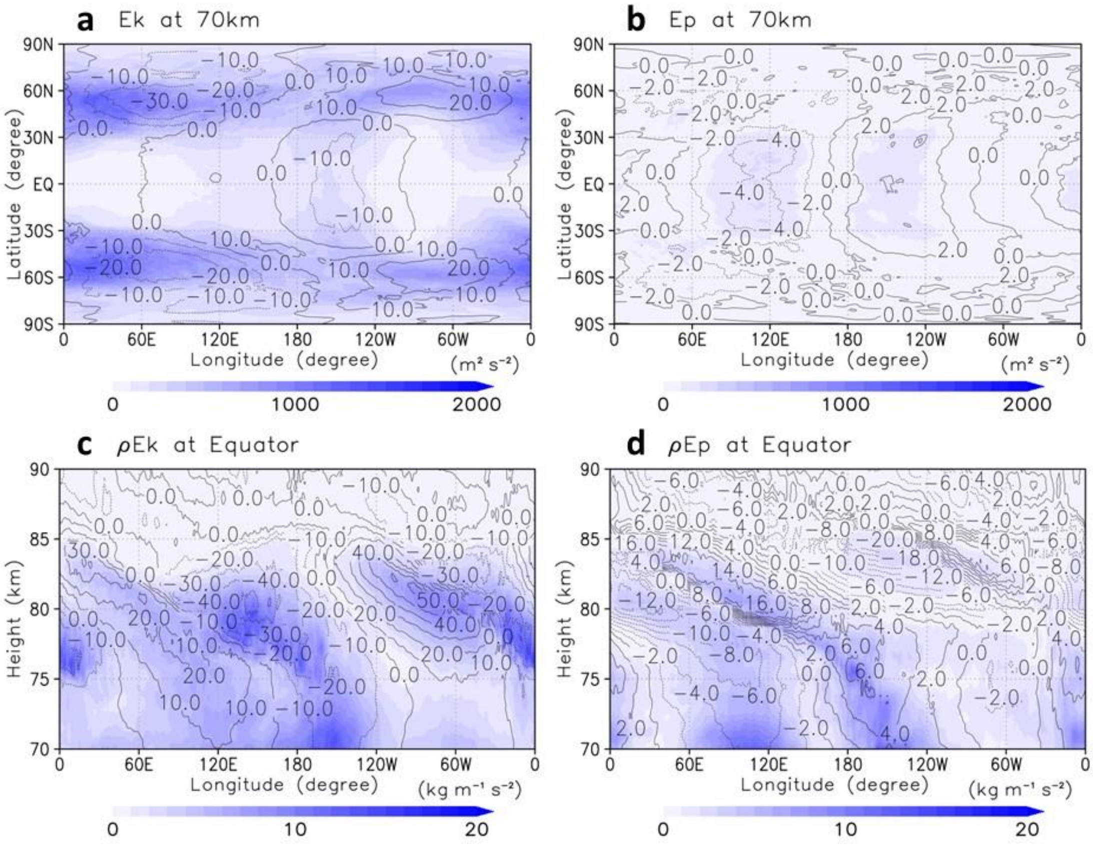

Supplementary Figure 2 Eddy kinetic energy and potential energy for the nominal case. Panels **a**, **b** show composite-mean eddy kinetic energy E_k (colour shades, $m^2 s^{-2}$) and potential energy E_p (colour shades, $m^2 s^{-2}$) in longitude–latitude cross-sections at the cloud-top (~70 km) level, respectively. Panels **c**, **d** shows density weighted composite-mean eddy kinetic energy ρE_k (colour shades, kg $m^{-1} s^{-2}$) and potential energy ρE_p (colour shades, kg $m^{-1} s^{-2}$) in longitude–height cross-sections at the equator, respectively. Disturbances of zonal flow (black contours; intervals are 10 m s^{-1}) and temperature (black contours; intervals are 2 K) from their zonal averages are also shown for **a**, **c** and **b**, **d**, respectively. The subsolar point is located at the centre of each panel, (180˚E, 0˚N).

Supplementary Figure 3 shows eddy kinetic energy E_k and potential energy E_p obtained for the Qz case. Because of lack of thermal tides, large amount of E_k and E_p were almost zonally uniform and concentrated in the mid- and high-latitudes. Amount of E_k and E_p were very small in the low-latitudes. It is clear that there were almost no small-scale gravity waves or their sources in the low-latitudes for Qz. These results strongly suggested that the small-scale gravity waves observed for the nominal case were generated from thermal tides.

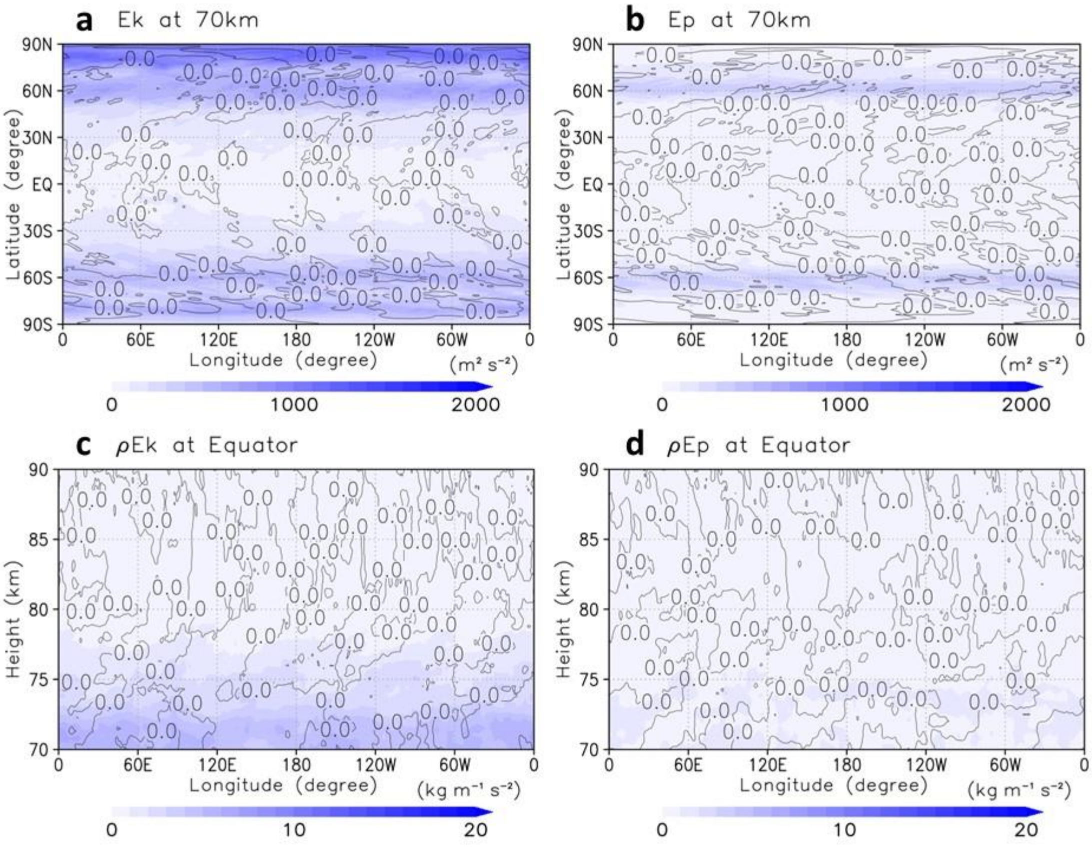

Supplementary Figure 3 As in Supplementary Figure 2 but for the Qz case.

<u>Supplementary Note 4 Static stability and Richardson number</u>

Supplementary Figure 4 shows snap shot of static stability and Richardson number obtained for the nominal case. Regions with small static stability (~80km, 120 ˚E–180˚E) correspond to those with small Richardson number. However, Regions with large temperature fluctuations (contour) caused by small-scale gravity waves do not correspond to those with small static stability and small Richardson number, suggesting that small scale gravity waves are not generated from unbalanced instability (Kelvin-Helmholtz

shear instability) at least in the present results.

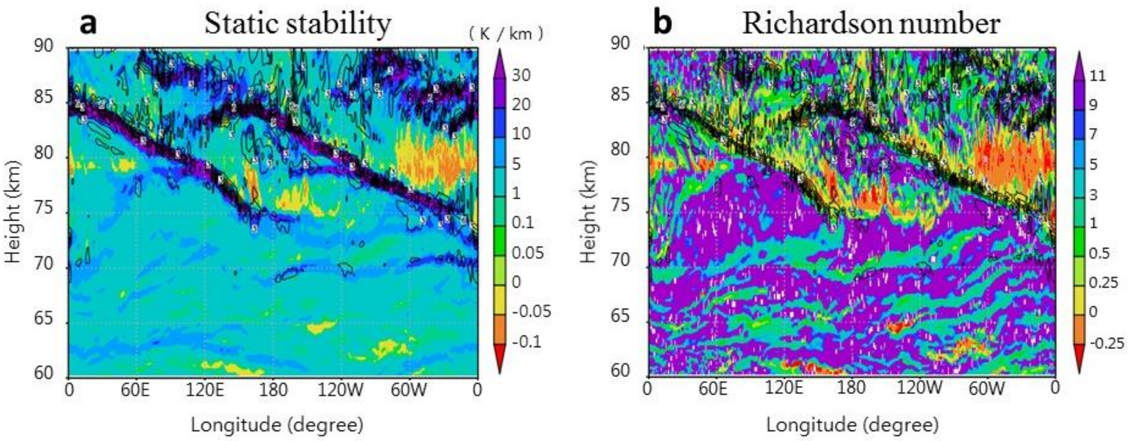

Supplementary Figure 4 Static stability and Richardson number for the nominal case. Panels **a**, **b** show static stability (colour shades; K km^{-1}) and Richardson number (colour shades) in longitude–height cross-sections at the equator, respectively. Disturbances of temperature (black contours; intervals are 3 K) from its zonal average are also shown. The subsolar point is located at the centre of each panel, (180˚E, 0˚N).

Supplementary Note 5 Vertical momentum flux and its mean acceleration of the super-rotation for the Qz case.

Supplementary Figure 5 shows vertical momentum flux $\overline{u'w'}$ in the longitude–latitude cross-section at the cloud-top level and mean zonal acceleration of the super-rotation due to the vertical momentum flux expressed by Eq. (4) in the longitude–height cross-section at the equator. The small-scale gravity waves were spontaneously radiated from

barotropic/baroclinic waves from the mid- to high-latitudes regions (Supplementary Figure 5a; the counterpart to Figure 4a). Compared with the nominal case, the small-scale structures were almost zonally uniform. There were no regions with large momentum flux in the low-latitudes and its mean zonal acceleration of the super-rotation in the vertical section at the equator (Supplementary Figure 5b; the counterpart to Figure 5a). It is again clear that there were almost no small-scale gravity waves or their sources for the Qz case in the low-latitudes. It is strongly suggested that the small-scale gravity waves obtained for the nominal case were generated from thermal tides, at least in the low-latitudes.

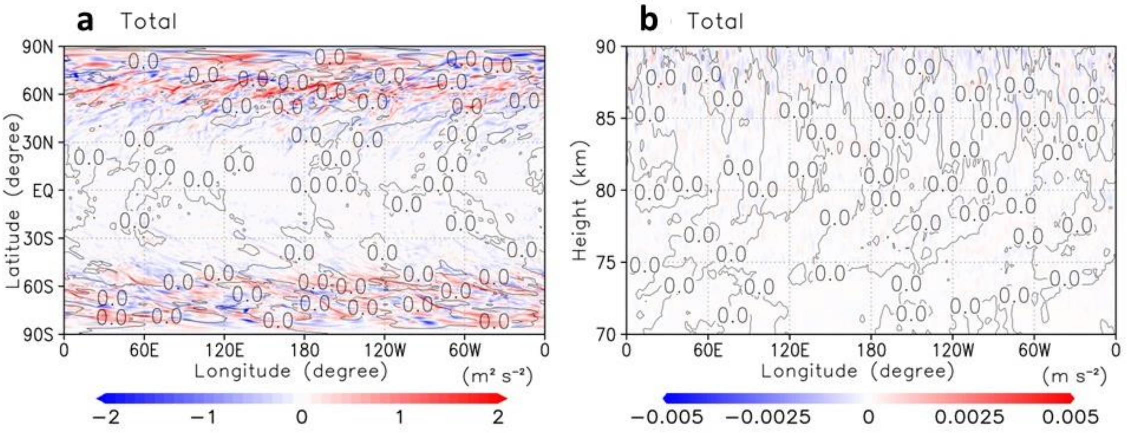

Supplementary Figure 5 Vertical momentum flux and its mean zonal forcing for the Qz case. Panels **a**, **b** show composite-mean vertical momentum flux (colour shades, m^2 s^{-2}) in the longitude–latitude cross-section at the cloud-top (~70 km) level and its mean zonal forcing (colour shades, m s^{-2}) in the longitude–height cross-section at the equator, respectively. Zonal flow disturbances from its zonal average (black contours; intervals

are 10 m s^{-1}) are also shown.

Supplementary Note 6 The coefficient of Rayleigh friction used as the sponge layer.

Supplementary Figure 6 shows vertical profile of the coefficient of Rayleigh friction used as the sponge layer, which are given by Eq. (5). It increases with height and acts effectively greater than ~100 km. A relaxation time of 0.05 Earth day is set at 120 km. We have checked dependency on the coefficient of the sponge layer in the previous studies[3] and confirmed that there was no significant changes in general circulation with different values of the coefficient.

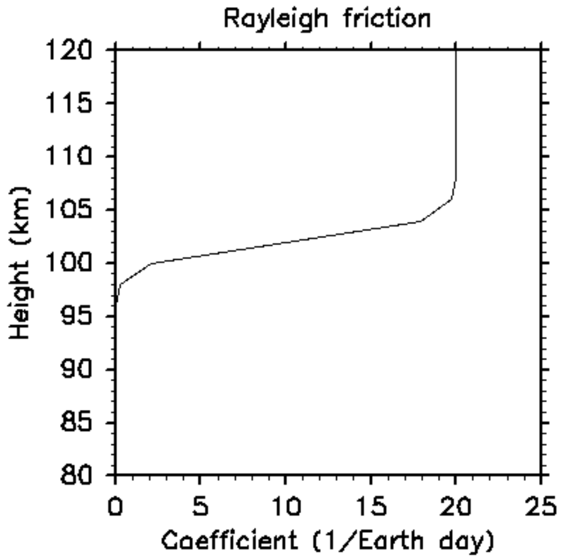

Supplementary Figure 6 The coefficient of Rayleigh friction. Vertical profile of the coefficient of Rayleigh friction (1/Earth day) used as the sponge layer is shown. At 120 km, e-folding time is 0.05 Earth days.

Supplementary References

1. Yamamoto, M. & Takahashi, M. Superrotation and equatorial waves in a T21 Venus-like AGCM. *Geophys. Res. Lett*. **30**(9):1449 (2003).

2. Sugimoto, N., Takagi, M. & Matsuda, Y. Fully developed super-rotation driven by the mean meridional circulation in a Venus GCM. *Geophys. Res. Lett.* **46**, 1776–1784 (2019).

3. Sugimoto, N., Takagi, M. & Matsuda, Y. Baroclinic instability in the Venus atmosphere simulated by GCM. *J. Geophys. Res.* **119**, 1950–1968 (2014).
