# Supplementary material for: Generation of gravity waves from thermal tides in the Venus atmosphere
Source: Nat Commun. 2021 Jun 17;12:3682. doi: 10.1038/s41467-021-24002-1 (PMC8211692; doi:10.1038/s41467-021-24002-1)
Supplement: Supplementary file 3 — Description of Additional Supplementary Files [file 41467_2021_24002_MOESM3_ESM.docx]

**Description of Supplementary Files**

**File Name: Supplementary Movie 1**

**Description:** The temporal variations of vertical wind velocity (colour, m s−1) in longitude–latitude cross-sections at the cloud-top level for the nominal case. The direction of planetary rotation in AFES-Venus is the same as that of Earth; then the super-rotation directs from west to east as described in Methods. Geopotential height disturbances from their zonal averages (black contours; intervals are 1000 m2 s–2 and dotted contours indicate negative values) are also shown. Data output interval is 1 hour. The subsolar point is located at (176.8˚W, 0˚N) at the beginning of animation.

**File Name: Supplementary Movie 2**

**Description:** Same as Supplementary Movie 1 but for the Qz case.

**File Name: Supplementary Movie 3**

**Description:** The temporal variations of vertical wind velocity (colour, m s−1) in longitude–height cross-sections at the equator for the nominal case. The direction of planetary rotation in AFES-Venus is the same as that of Earth; then the super-rotation directs from west to east. Geopotential height disturbances from their zonal averages (black contours; intervals are 1000 m2 s–2 and dotted contours indicate negative values) are also shown. Data output interval is 1 hour. The subsolar point is located at (176.8˚W, 0˚N) at the beginning of animation.

**File Name: Supplementary Movie 4**

**Description:** Same as Supplementary Movie 3 but for the Qz case.
