# Supplementary figures and images for: Generation of gravity waves from thermal tides in the Venus atmosphere
Source: Nat Commun. 2021 Jun 17;12:3682. doi: 10.1038/s41467-021-24002-1 (PMC8211692; doi:10.1038/s41467-021-24002-1)

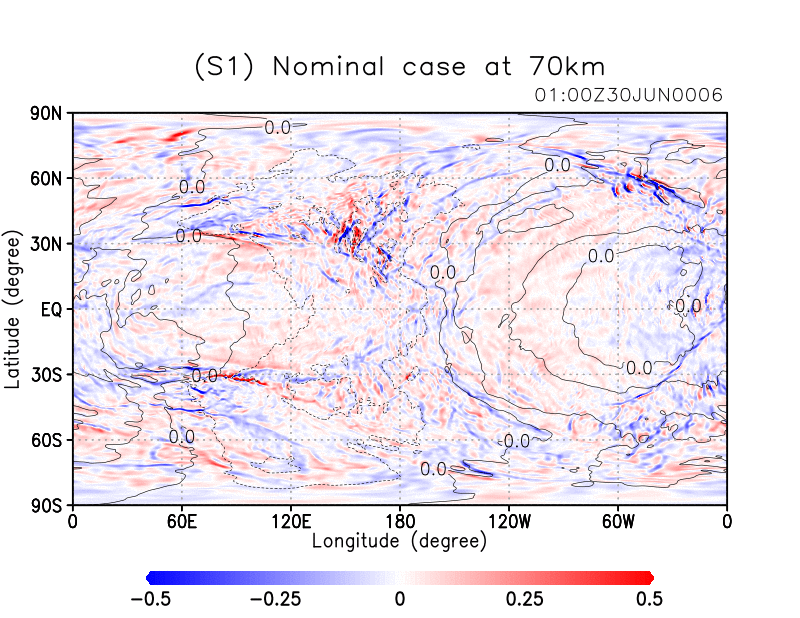

Supplement: Supplementary file 4 — Supplementary Movie 1 [file 41467_2021_24002_MOESM4_ESM.gif]

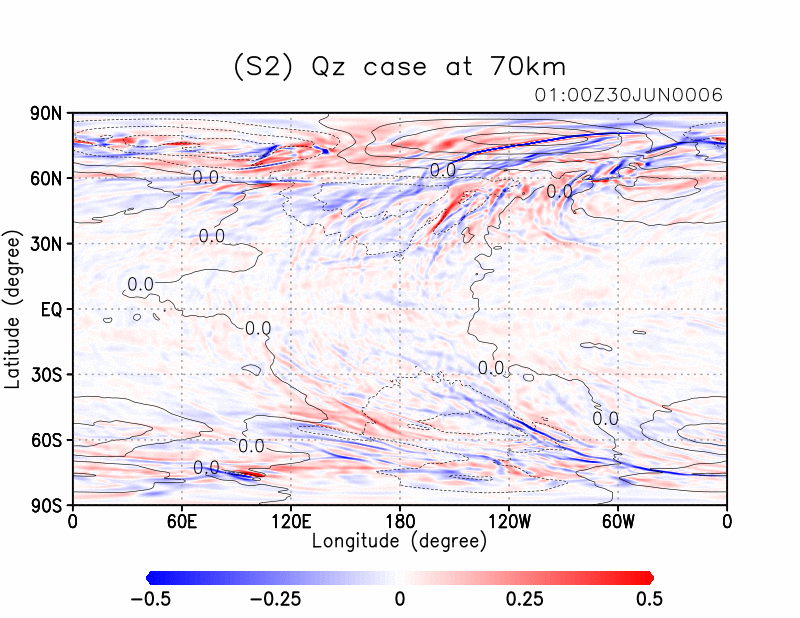

Supplement: Supplementary file 5 — Supplementary Movie 2 [file 41467_2021_24002_MOESM5_ESM.gif]

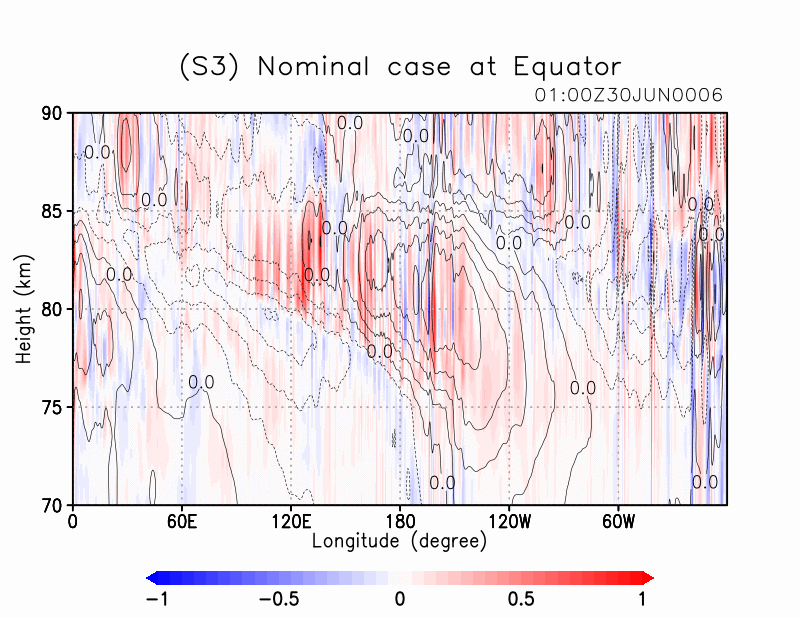

Supplement: Supplementary file 6 — Supplementary Movie 3 [file 41467_2021_24002_MOESM6_ESM.gif]

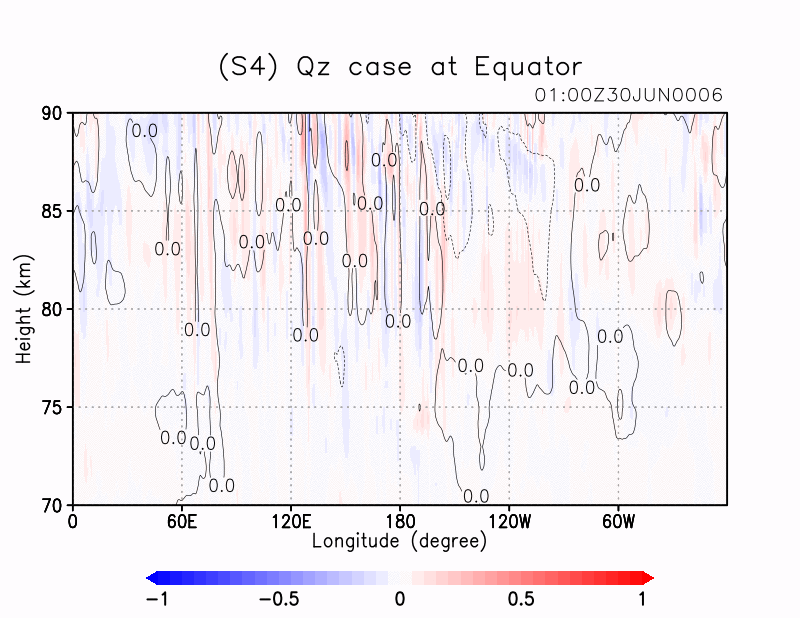

Supplement: Supplementary file 7 — Supplementary Movie 4 [file 41467_2021_24002_MOESM7_ESM.gif]
